# mid-IR dataset from low molecular weight permeate of ultra-filtered spent sulfite liquor

**DOI:** 10.1016/j.dib.2024.110752

**Published:** 2024-07-18

**Authors:** Yannick Bus, Daniel Waldschitz, Oliver Spadiut

**Affiliations:** Research Group Bioprocess Technology, Institute of Chemical, Environmental and Bioscience Engineering, TU Wien, Gumpendorferstraße 1A, A-1060 Vienna, Austria

**Keywords:** Lignocellulosic feedstock, In-line spectroscopy, Raw material variation, PAT

## Abstract

The dataset consists of FTIR spectra of ultra-filtered spent sulphite liquor (UF-SSL) from softwood pulping obtained from one paper mill biorefinery plant with the purpose of *real-time* quantification of the sugar content of UF-SSL. Data collection was performed using a submerged mid-IR probe placed in a continuously stirred tank reactor and reference sugar measurements were performed using HPLC. Spectra were obtained of raw and spiked UF-SSL. As “low complexity” case 25% UF-SSL from one batch was analysed for its 3 most abundant sugars (mannose, xylose, glucose) and as “high complexity” case 25/50/75% UF-SSL from 2 batches was analysed for its 5 most abundant sugars (the latter + galactose, arabinose). In both cases, independent single sugar spikes and simultaneous multiple sugar spikes were performed. Real time *in-line* data was generated by stepwise and gradual changes in sugar composition over time with a run time of >200 h.

Specifications Table

**Table:** 

Subject	Analytical Chemistry: Spectroscopy
Specific subject area	Spectroscopic analysis of lignocellulosic process side streams from forestry residues with a focus on sugars for use in biotechnology.
Type of data	Table Raw, Analyzed .xslx file
Data collection	The FT-MIR spectra were taken by a Fiber MultiplexIR FT-IR system (ReactIR 45 m, Mettler Toledo, USA) equipped with a liquid N2 MCT detector and an optical fiber (1.5 m) immersion probe from silver halide, with 9.5 mm optical path length and a DiComp diamond probe tip (ReactIR 45 m, Mettler Toledo, USA). Each spectrum (3000 cm^−1^ to 650 cm^−1^) and consisted of an average of 256 scans, with a resolution of 4 cm^−1^. Sugar reference measurements were measured off-line by HPLC (Ultimate 3000, Thermo Fisher Scientific, USA) equipped with an RI detector (RI100, Shodex, USA) using a Pb-column (NUCLEOGEL SUGAR Pb719530, Machery-Nagel, Germany) at 79 °C with an isocratic flow of 0.4 ml/min ultra-pure.
Data source location	Research Area Bioprocess Engineering, Institute of Chemical, Environmental and Bioscience Engineering, TU Wien, Gumpendorferstraße 1A, A-1060 Vienna, Austria
Data accessibility	Repository name: Mendely Data Data identification number: doi: 10.17632/62zrbmgh2v.1 Direct URL to data: https://data.mendeley.com/datasets/62zrbmgh2v/1
Related research article	WALDSCHITZ, Daniel, et al. Addressing raw material variability: In-line FTIR sugar composition analysis of lignocellulosic process streams. Bioresource Technology, 2024, 399. Jg., S. 130535.

## 1 Value of the Data

• The dataset is useful in quantifying the impact of monosaccharides as well as the impact of the background on the mid-IR spectra within the complex matrix of spent sulfite liquor.
• Raw spectra and sugar reference values of samples are provided for model calibration and validation, applicable for multiple linear regression and partial least square regression model building by researchers.
• Spectroscopic data of prolonged (1 spectrum every 3 min for >200 h) *in-line* use with reference measurements every 1.5–3 h can be used to evaluate the performance of models developed by researchers with gradual and step-wise changes in background levels and sugar composition.
• The presented data is beneficial to pulp and paper mill operators seeking a more sophisticated, real-time understanding of the variability in the composition of their mill side stream. Further, it is relevant to biotechnologists working on the valorization of this renewable side stream for the production of renewable fuels, such as bioethanol, platform chemicals or feed additives, such as single cell protein. The growth and production capabilities of microorganisms are critically influenced by the composition of the renewable feedstock, especially the C5/C6 sugar ratio [1]. Therefore, the dataset can help researchers to better understand the innate variability of renewable feedstocks, to improve cultivation procedures and thereby increase the economic feasibility of their circular economy processes.

## 2 Background

The primary objective for generation of the dataset was to evaluate mid-IR spectroscopy for real time quantification of sugars in spent sulfite liquor (SSL), as current reference methods are time consuming. Currently, most of the SSL is incinerated, where its exact composition is irrelevant. However, due to its abundance of metabolizable sugars, biotechnological applications are often proposed, for which the composition and concentration of sugars are of high relevance. Mid-IR was used to analyze ultra-filtered SSL as the carbohydrate region of fundamental molecular vibrations lies within the mid-IR spectral range. This data article adds value to the published dataset [2] and the research article [3] by including spectra and spectral regions not analyzed in the research article, as well as providing in-depth information on the sample preparation process aimed at researchers for generation of their own independent evaluation of the data.

## 3 Data Description

The dataset is structured in a so-called “low complexity” case (25% UF-SSL of 1 batch analyzed for mannose, xylose and glucose concentration) and a “high complexity” case (25/50/75/100% UF-SSL from 2 batches analyzed for mannose, xylose, glucose, galactose and arabinose concentration). Each case is structured as a calibration set (1 sugar altered at a time), a validation set (multiple sugars altered simultaneously) an *in-line* reference set (in-line spectra at time points of reference measurements) and all in-line spectra (every 3 min for > 200 h) as shown in Table 1.

**Table 1:** Dataset structure within the .xlsx file.

Worksheet	Samples	Reference Data	Spectra
LowComplexityCalibartionSet	32	Glucose, Mannose, Xylose in g/L	3000–650 cm^−1^
LowComplexityValidationSet	14	Glucose, Mannose, Xylose in g/L	3000–650 cm^−1^
LowComplexityInlineReference	76	Glucose, Mannose, Xylose in g/L	3000–650 cm^−1^
LowComplexityInlineAll	5201		3000–650 cm^−1^
HighComplexityCalibartionSet	205	Glucose, Xylose, Galactose, Arabinose, Mannose in g/L	3000–650 cm^−1^
HighComplexityValidationSet	112	Glucose, Xylose, Galactose, Arabinose, Mannose in g/L	3000–650 cm^−1^
HighComplexityInlineReference	224	Glucose, Xylose, Galactose, Arabinose, Mannose in g/L	3000–650 cm^−1^
HighComplexityInlineAll	4779		3000–650 cm^−1^

Calibration set samples are labeled by %UF-SSL background, sugar spiked and level (e.g. SSL25_GLC_9). Validation set samples are labeled by %UF-SSL background, set number and level (e.g. SSL50_1_4) as multiple sugars were spiked simultaneously. In-line samples are labelled by process time in hours when the spectra were recorded.

## 4 Experimental Design, Materials and Methods

### 4.1 Solution preparation

The samples used and measured for calibration, validation and application of the models, were prepared from UF-SSL batches (stored at 4 °C). For that, base solutions of 25 % UF-SSL mixed with water were prepared. Furthermore, UF-SSL solutions with higher concentrations of UF-SSL were prepared containing 25 %, 50 % and 75 % UF-SSL. Additionally, also solutions containing higher concentrations of one specific sugar were prepared. Mannose, Xylose, Glucose, Galactose and Arabinose, respectively, were added to UF-SSL. The solutions were prepared volumetrically from a well-mixed batch of UF-SSL. All solutions were well mixed and subsequently filtered with a sterile bottle top filter (0.2 µm). Samples of all solutions were analyzed for sugar concentration with HPLC and stored at 4 °C until use.

### 4.2 Sample preparation for calibration and validation

The samples for calibration and validation were prepared in a stirred (150 rpm) glass vessel with 1 L working volume and kept at 20 °C with a water jacket. 500 mL of the UF-SSL base solution was filled in the vessel and was then constantly stirred. To increase the sugar concentrations from +1% to +28% sugar relative to amount of the respective sugar in 100% UF-SSL, the UF-SSL spike solution, containing high concentrations of one specific sugar, were added with the required volume. After 60 seconds of constant stirring the mixture of base solution and spike solution was measured with mid-IR and a 2 mL sample was drawn. The sample were stored at 4 °C until further analysis.

### 4.3 Sample preparation for in-line application

The samples were prepared and measured in a 3.5 L glass vessel, that was stirred at 150 rpm speed, kept at 20 °C and included top gassing with N_2_. 1.4 L of base solution of 25 % UF-SSL was filled in as the first concentration step. A UF-SSL solution with either a different UF-SSL concentration or a different concentration of a specific sugar was connected through a tube and a pump to the vessel and was filled after the end of the first concentration step. Two different procedures of adding UF-SSL were carried out. Either everything was added immediately (<90 sec) or it was added constantly with a constant flow rate. Upon reaching the maximum working volume of 3 L, UF-SSL was removed until 1.4 L remained inside the glass vessel. The addition of more UF-SSL solutions with various sugar concentrations and UF-SSL concentrations and the drainage of the full vessel was carried on until the end of the in-line application.

During each concentration step, samples were taken automatically with a Numera Secure Cell (Switzerland) connected through a tube inside the vessel. The intervals between each sample were between 1 – 3 hours. The samples were stored at 4 °C until further analysis.

### 4.4 FTIR spectra acquisition

The FT-MIR spectra of the prepared sample were collected in the glass vessels with a Fiber MultiplexIR FT-IR system (ReactIR 45 m, Mettler Tolido, USA) equipped with a liquid N_2_ MCT detector and an optical fiber immersion probe from silver halide, with 9.5 mm optical path length and a DiComp diamond probe tip (ReactIR 45 m, Mettler Tolido, USA) which was connected with a 1.5 m long fibre optic cable. To have as little bending in the fibre optic cable as possible, the same measurement configuration was kept for all experiments, and the cable was fixated with a clamp. The probe was inserted through a port on top of the glass vessels to stably fixed while acquiring spectra inside the vessel. Before taking the first sample spectrum a background spectrum had to be taken with the specs mentioned before. For that, the probe was placed in the same position as during spectra acquisition of a sample while the vessel was still empty (probe tip surrounded only by air).

Each spectrum ranged from 3000 cm^−1^ to 650 cm^−1^ and consisted of an average of 256 scans, with a resolution of 4 cm^−1^. For calibration and validation samples, the spectrum with an average of 256 scans was acquired by manually initiating the spectra acquisition once the media was well mixed. During the in-line application, spectra were taken and saved automatically every three minutes for the duration of the experiment.

### 4.5 HPLC offline measurement

The reference values for each sample were measured with HPLC. For that Glucose, Xylose, Arabinose, Galactose and Mannose were measured off-line by HPLC (Ultimate 3000, Thermo Fisher Scientific, USA) equipped with an RI detector (RI100, Shodex, USA) using a Pb-column (NUCLEOGEL SUGAR Pb 719530, Machery-Nagel, Germany) at 79 °C with an isocratic flow of 0.4 ml/min ultra-pure water with a runtime of 65 min. All samples were diluted 1:20 with ultra-pure water and filtered using a 0.22 um filter before placing them on the instrument.

## Limitations

The sugar composition of SSL is known to be affected by the wood type (tree species) and pulping technique used [4]. The UF-SSL used for creation of this dataset was obtained from a single paper mill within an integrated biorefinery context. Hence, solely SSL from soft wood pulping of spruce was used for spectroscopic analysis.

## Ethics Statement

The described data does not involve human subjects, animal experiments, or any data collected from social media platforms and follows all ethical requirements.

## CRediT authorship contribution statement

**Yannick Bus:** Formal analysis, Investigation, Methodology, Validation, Data curation, Writing – original draft. **Daniel Waldschitz:** Formal analysis, Investigation, Project administration, Data curation, Visualization, Writing – original draft. **Oliver Spadiut:** Supervision, Writing – review & editing.

## Data Availability

mid-IR raw spectra from low molecular weight permeate of ultra filtrated spent sulfite liquor (UF-SSL) including sugar reference measurements (Original data) (Mendeley Data).

## References

[1] Kenney K.L. (2013). Understanding biomass feedstock variability. Biofuels.

[2] Waldschitz D., Bus Y. (2023). mid-IR raw spectra from low molecular weight permeate of ultra filtrated spent sulfite liquor (UF-SSL) including sugar reference measurements. Mendeley Data.

[3] Waldschitz D. (2024). Addressing raw material variability: In-line FTIR sugar composition analysis of lignocellulosic process streams. Bioresource Technol..

[4] Fatehi P., Ni Y. (2011). Sustainable Production of Fuels, Chemicals, and Fibers from Forest Biomass.

